# Intravenously Administered Human Umbilical Cord-Derived Mesenchymal Stem Cell (HucMSC) Improves Cardiac Performance following Infarction via Immune Modulation

**DOI:** 10.1155/2023/6256115

**Published:** 2023-03-17

**Authors:** Xiaoting Liang, Jing Liu, Mimi Li, Fang Lin, Rulin Zhuang, Qingshu Meng, Xiaoxue Ma, Yuanfeng Xin, Xin Gong, Zhiying He, Wei Han, Xiaohui Zhou, Zhongmin Liu

**Affiliations:** ^1^Research Center for Translational Medicine, Shanghai East Hospital, Tongji University School of Medicine, Shanghai 200120, China; ^2^Institute for Regenerative Medicine, Shanghai East Hospital, School of Life Sciences and Technology, Tongji University, Shanghai 200120, China; ^3^Department of Burn & Plastic Surgery, Beijing Children's Hospital, Capital Medical University, National Center for Children's Health, Beijing 100045, China; ^4^Shanghai Heart Failure Research Center, Shanghai East Hospital, Tongji University School of Medicine, Shanghai 200120, China; ^5^Department of Cardiovascular Surgery, Shanghai East Hospital, Tongji University School of Medicine, Shanghai 200120, China; ^6^Department of Heart Failure, Shanghai East Hospital, Tongji University School of Medicine, Shanghai 200120, China; ^7^Shanghai Institute of Stem Cell Research and Clinical Translation, Shanghai 200120, China

## Abstract

Overactive inflammatory responses contribute to progressive cardiac dysfunction after myocardial infarction (MI). Mesenchymal stem cell (MSC) has generated significant interest as potent immune modulators that can regulate excessive immune responses. We hypothesized that intravenous (iv) administration of human umbilical cord-derived MSC (HucMSC) exerts systemic and local anti-inflammation effects, leading to improved heart function after MI. In murine MI models, we confirmed that single iv administration of HucMSC (30 × 10^4^) improved cardiac performance and prevented adverse remodeling after MI. A small proportion of HucMSC is trafficked to the heart, preferentially in the infarcted region. HucMSC administration increased CD3^+^ T cell proportion in the periphery while decreased T cell proportion in both infarcted heart and mediastinal lymph nodes (med-LN) at 7-day post-MI, indicating a systematic and local T cell interchange mediated by HucMSC. The inhibitory effects of HucMSC on T cell infiltration in the infarcted heart and med-LN sustained to 21-day post-MI. Our findings suggested that iv administration of HucMSC fostered systemic and local immunomodulatory effects that contributed to the improvement of cardiac performance after MI.

## 1. Introduction

Myocardial infarction (MI), caused primarily by occlusion of the coronary vessels, represents the most common cause of mortality worldwide. Over 70% of patients developed progressive adverse left ventricular (LV) remodeling and eventual heart failure despite optimal medical care [1]. Recently, the overactive post-MI inflammatory response has been proposed as an essential contributor to progressive myocardial dysfunction [2–4]. Strategies targeting postinfarction inflammation may exert beneficial actions by attenuating adverse remodeling after infarction. However, broad inhibition of the inflammatory cascade potentially impairs the reparation process in the infarcted heart [5]. Therefore, timely and environmental-adaptive immune regulation may be an optimal strategy to orchestrate cardiac healing and remodeling post-MI.

Most recently, the immunomodulatory effects of mesenchymal stem cells (MSC) have attracted considerable attention in MI treatment [6]. MSC, a fibroblast-like, multipotent heterogeneous population, can be isolated from many sources, such as bone marrow, umbilical cord, muscle, peripheral blood, synovial membrane, and adipose tissue [7]. Among them, umbilical cords provide a prosperous pool for fetal cells that possess multipotent properties, and MSC derived from the umbilical cord present comparatively greater proliferative and immunosuppressive capacities compared to those derived from adult tissues [8, 9]. Previous studies revealed that MSC can regulate the activation and function of various cell types after infarction by releasing multiple mediators, including immunosuppressive molecules, growth factors, exosomes, chemokines, and metabolites [10]. On the other hand, different immune microenvironments affect the performance of MSC, indicating that the immunoregulatory function of MSC is highly plastic [6]. For instance, MSC administration in the inflammatory phase successfully ameliorated the graft-versus-host disease (GvHD). However, when MSC was transplanted before the inflammation, they cannot exert their efficacy very well [11]. Such plasticity in MSC immunoregulation empowers them as a potential candidate to deliver appropriate modulation of inflammatory responses.

The inflammatory response after MI not only involves the immune cells residing in the heart but also refers to the recruitment and mobilization of the immune cells throughout the body, such as the patrolling pool in the peripheral blood and the splenic reservoir [12]. Based on this concept, intravenous- (iv-) administered MSC could exert systemic and local anti-inflammation effects, leading to improved heart function after MI. A previous study demonstrated that intravenous delivery of bone marrow MSC (BM-MSC) significantly decreased the ratio of natural killer cells and neutrophils in the heart and attenuated the progressive deterioration in LV function and adverse remodeling in a mouse model of ischemia and reperfusion [2]. However, since the acquisition procedure of BM-MSC was invasive, with a significant decrease in the yield and proliferative capacity with the donor's age, exploiting potential alternative sources could be beneficial [13, 14]. In addition, MSC isolated from different origins exhibits a considerable degree of variabilities in terms of proliferation potentials and immunomodulation capacities [15, 16]. It was reported that MSC isolated from fetal or close to fetal sources with relatively undifferentiated phenotype showed advantages over MSC isolated from adult sources [17]. In light of this, the umbilical cord is an attractive source of MSC, as it can be harvested noninvasively at a low cost, especially with their abundant availabilities, relatively primitive status, and high proliferation potential. MSC isolated from the umbilical cord showed the fastest population doubling time compared to those isolated from other adult tissues [18]. We recently demonstrated that intramyocardial delivered human umbilical cord-derived MSC (HucMSC) contributed to cardiac function recovery by promoting CD4^+^ T cell migration into the infarcted heart [19]. However, little is known about the therapeutic value of iv HucMSC post-MI and whether their immune regulatory effects played a role during this process. Thus, this study evaluates the efficacy of iv-administered umbilical cord MSC on cardiac functional recovery and their immunomodulatory effects in a murine model of MI.

## 2. Material and Methods

### 2.1. Ethics Statement

All animal experiments were approved by the Committee on the Use of Live Animals in Teaching and Research of the Tongji University for Laboratory Animal Medicine (approval no: TJLAC-019-131). The human umbilical cords were obtained from full-term births and implemented following the approval of the Ethics Committee and the informed consent of donors in Shanghai East Hospital.

### 2.2. HucMSC Culture and Characterization

HucMSC was isolated from the umbilical cord and cultured as previously described [19, 20]. Briefly, the umbilical cord was cut into 2-3 cm pieces, and the vessels were removed. Wharton's jelly was separated and cut into 1 cm pieces. Then, the tissues were placed onto the culture dish and cultured in minimum essential medium alpha (*α*-MEM, Corning, 10-022) supplemented with 5% UltraGRO-Advanced (Helios, HPCFDCGL50). The medium was changed every 2-3 days. HucMSC was characterized by surface marker profiling and trilineage differentiation capacity as previously described [19]. HucMSC at passages 4-6 was used in the current study.

### 2.3. DiR-Labeled HucMSC

HucMSC was prestained with the near-infrared fluorescent-lipophilic tracer 1,1-dioctadecyl-3,3,3,3-tetramethylindotricarbocyanine iodide (DiR, Thermo Fisher, D12731) according to the manufacturer's instructions. To identify tissue-engrafted HucMSC following intravenous injection, mice were sacrificed at days 7 and 28 post-MI. The multiple organs of murine (including heart, lung, liver, kidney, and spleen) were harvested and analyzed using the IVIS imaging instrument (Xenogen, USA) with the relevant channel.

### 2.4. MI Model

The MI model was induced in adult C57BL/6 J mice (6–8 weeks) by permanent ligation of the left anterior descending coronary artery with an 8-0 silk suture as previously described [21]. At approximately 1 hour after MI (after recovery from anesthesia), the mice were randomly assigned (using computer-generated random numbers) to the following treatments: (1) tail vein injection of 200 *μ*L saline (MI group, *n* = 12); (2) tail vein injection of 30 × 10^4^ HucMSC suspended in 200 *μ*L saline (MI-HucMSC group, *n* = 12). Cardiac performance was evaluated by transthoracic echocardiography (Ultramark 9; Soma Technology, Bloomfield, CT, USA) at baseline (before MI), 7 days and 28 days following MI. M-mode images were utilized to calculate LV function, including LV ejection fraction (LVEF) and LV fraction shortening (LVFS).

### 2.5. Enzyme-Linked Immunosorbent Assay (ELISA)

Serum was collected at baseline, 7-day and 28-day post-MI. The serum ANP (atrial natriuretic peptide) and BNP (brain natriuretic peptide) levels were determined by ELISA as per the manufacturer's instructions (Shanghai Jianglai Industrial, JL20612-96 T for ANP, JL12884-96 T for BNP).

### 2.6. Masson's Trichrome Staining

Heart tissues were harvested at 28-day post-MI. Masson's trichrome staining was performed to assess cardiac fibrosis as previously described [22]. The percentage of infarct size was determined as infarcted areas/total LV areas ×100% and measured using K-Viewer software (KFbio, China).

### 2.7. Heart Weight Index Calculation

The body weight of each mouse was measured before sacrificing the animals. The heart weight (without heart auricles, pericardium, and blood) was recorded after carefully explanting the organ. The heart weight index (HWI) was calculated as heart weight (mg)/body weight (g).

### 2.8. Wheat Germ Agglutinin Staining

The paraformaldehyde-fixed, paraffin-embedded heart sections were deparaffinized and rehydrated according to the standard procedure as previously described [23, 24]. Briefly, after antigen retrieval and blocking, the slides were stained with wheat germ agglutinin (WGA) conjugated to Alexa Fluor 488 (Thermo Fisher, W11261) overnight, and the nucleus was counterstained with 2-(4-Amidinophenyl)-6-indolecarbamidine dihydrochloride (DAPI). The fluorescent signals were visualized and captured with a Leica DM6000B microscope. Five high-power fields were randomly selected for quantifications of myocyte cross-sectional area using ImageJ software (version 1.53 k, National Institutes of Health, Bethesda, MD, USA). A minimum of 200 myocytes from 5 to 6 different animals was quantified for each experimental group.

### 2.9. Immunofluorescence Staining

Mouse tissue was paraformaldehyde fixed, paraffin embedded, and sectioned for standard immunostaining as previously described [25, 26]. The information of the primary antibodies was as follows: anti-CD31 (1 : 100, cell signaling, 3528S), anti-CD3 (1 : 100; Abcam, ab231775), and anti-CD45 (1 : 100, cell signaling, 70257 s). The mean vessel density was calculated as vessel area (CD31 positive)/total area × 100% using ImageJ software (version 1.53 k, National Institutes of Health, Bethesda, MD, USA). Five high-power fields from 5 to 6 different animals in each group were randomly selected for quantifications.

### 2.10. Quantitative Reverse Transcription Polymerase Chain Reaction

Total RNA from heart tissue, including infarct and peri-infarct area, was isolated with TRIzol reagent (Beyotime, R0016). The cDNA was synthesized from 500 ng of total RNA with a RevertAid First Strand cDNA Synthesis Kit (Takara, RR0036A). Quantitative PCR was performed using Fast SYBR Green Master Mix (Thermo Fisher, 4385617) in an ABI QuantStudio 6 Flex System. The relative standard curve method (*ΔΔ*Ct) was used to determine the relative mRNA expression, with *β-actin* as reference. The primer sequences were listed as follows: ANP (atrial natriuretic peptide) forward: GCTTCCAGGCCATATTGGAG, ANP reverse: GGGGGCATGACCTCATCTT; BNP (brain natriuretic peptide) forward: AGTCCTTCGGTCTCAAGGCA, BNP reverse: CCGATCCGGTCTATCTTGTGC; *α-*SMA (alpha smooth muscle actin) forward: GTCCCAGACATCAGGGAGTAA, *α-*SMA reverse: TCGGATACTTCAGCGTCAGGA; TNF*-α* (tumor necrosis factor alpha) forward: CCTGTAGCCCACGTCGTAG, TNF*-α* reverse: GGGAGTAGACAAGGTACAACCC; IL1*-β* (interleukin 1 beta) forward: GCAACTGTTCCTGAACTCAACT, IL1*-β* reverse: ATCTTTTGGGGTCCGTCAACT; IL10 (interleukin 10) forward: GCTCTTACTGACTGGCATGAG, IL10 reverse: CGCAGCTCTAGGAGCATGTG; IFN-*γ* (interferon gamma) forward: ACAGCAAGGCGAAAAAGGATG, IFN*-γ* reverse: TGGTGGACCACTCGGATGA; TGF*-β* (transforming growth factor beta) forward: CTCCCGTGGCTTCTAGTGC, TGF*-β* reverse: GCCTTAGTTTGGACAGGATCTG; *β-*actin forward: GGCTGTATTCCCCTCCATCG, *β*-actin reverse: CCAGTTGGTAACAATGCCATGT.

### 2.11. Preparation of Single-Cell Suspensions for Flow Cytometry

Single-cell suspensions for flow cytometry analysis were prepared as previously described [2]. Briefly, after anesthetization, blood was sampled from the eyes and leukocytes were isolated with Ficoll (Solarbio, R1017) according to the manufactures' instructions. Cells were filtered through a 40 *μ*m cell strainer. To obtain single-cell suspensions from heart tissue, mice were perfused with saline through the LV to clear the remaining blood. Heart tissues were cut into 1 mm pieces and digested twice in the C-tube with collagenase II (1.5 mg/mL) and DNAse in gentleMACS™ Tissue Dissociators (Miltenyi Biotec). The heart tissue was centrifuged at 37°C for 30 min at a speed of 200 rpm. After removing debris, single-cell suspension was collected by mashing the digested tissue through a 70 *μ*m strainer. For splenic and mediastinal lymph node (med-LN) cell preparation, spleens and med-LNs were isolated and mashed with the plunger of a 1 mL syringe through a 70 *μ*m strainer. The collected cells were lysed with red blood cell lysis buffer (eBioscience, 00-4333-57) according to the instructions. Then, the cells were filtered through a 40 *μ*m strainer, counted, and maintained on ice for further analysis.

### 2.12. Flow Cytometry

Cells were stained with the relevant antibodies in FACS buffer for 30 min at 4°C. Anti-mouse antibodies used for flow cytometry staining were anti-mouse CD45 (Clone I3/2.3, Biolegend), CD19 (Clone 1D3/CD19, Biolegend), CD3 (Clone 17A2, Biolegend), F4/80 (Clone BM8, Biolegend), and Ly-6G (Clone 1A8, Biolegend). Flow cytometry was performed on BD FACSAria II and analyzed with FlowJo software (version 10.7.1, FlowJo LLC, USA).

### 2.13. Protein Array

Serum was collected at 7-day post-MI. The inflammatory cytokine protein levels, including IFN-*γ*, IL-12p70 (interleukin-12p70), IL-13 (interleukin-13), IL1-*β*, IL-2 (interleukin-2), IL-4 (interleukin-4), IL-5 (interleukin-5), IL-6 (interleukin-6), TNF-*α*, GM-CSF (granulocyte-macrophage colony-stimulating factor), IL-10, IL-17A (interleukin-17A), and IL-18 (interleukin-18), were determined by protein array (Thermo Fisher, EPX110-20820-901, EPX01A-20614-901, EPX01A-26005-901, and EPX01A-26009-901) as per the manufacturer's instructions.

### 2.14. Statistical Analysis

Continuous variables are presented as mean ± standard deviation (SD). Comparison of variables between the two groups was performed using an unpaired Student's *t*-test. Survival curves were plotted using the Kaplan–Meier method, and the difference among groups was compared using the log-rank test. Statistical analysis was performed with SPSS 20.0 and GraphPad Prism 8.0 software. A test was considered statistically significant with a *p* value less than 0.05.

## 3. Results

### 3.1. Distribution and Effects of Iv-Administered HucMSC in Murine MI Models

**Figure 1:**
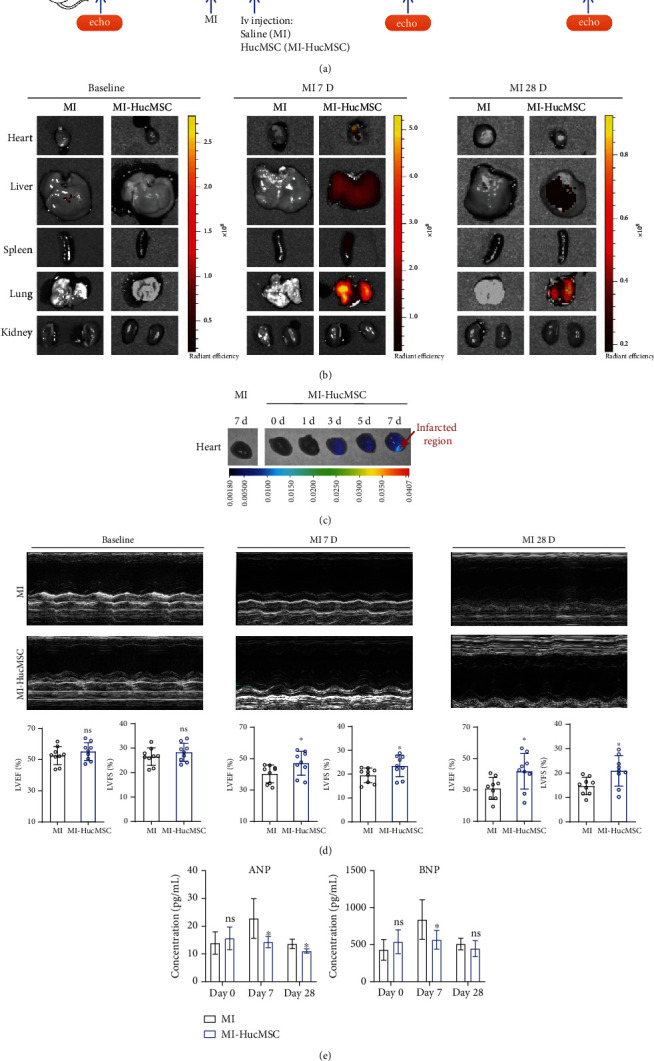
Distribution and effects of iv-administered HucMSC after infarction. (a) Schematic diagram of the study design. (b) Biodistribution of DiR-labeled HucMSC in different organs was measured at baseline, 7-day and 28-day post-MI. *n* = 3 mice for each group. (c) Biodistribution of DiR-labeled HucMSC in the heart at 0-, 1-, 3-, 5-, and 7-day post-MI. *n* = 3 mice for each group. (d) Representative echocardiography (upper panel) and calculation of LVEF and LVFS at baseline, 7-day and 28-day post-MI (lower panel). *n* = 8 ~ 9 mice for each group. (e) Serum ANP and BNP levels were measured at baseline, 7-day and 28-day post-MI by ELISA assay. *n* = 5 ~ 8 mice for each group. ns, non-significant vs. MI group; ^∗^*p* < 0.05 vs. MI group.

To confirm the phenotype and function of HucMSC, cells at passage 4 were characterized by surface marker profiling and trilineage differentiation capacity (supplementary Figure 1). HucMSC showed positive expression of MSC markers CD90, CD105, and CD73, and negative expression of hematopoietic markers CD11, CD34, and CD45 (supplementary Figure 1A). HucMSC showed their osteogenic, adipogenic, and chondrogenic differentiation potential (supplementary Figure 1B). To determine the in vivo distribution of HucMSC after intravenous injection, HucMSC was prestained by DiR and administered intravenously 1 hour after MI. The cardiac function was recorded at baseline (before MI), 7 days and 28 days after MI. A schematic diagram of the study design is shown in Figure 1(a). There was no significant change in survival rate between HucMSC-treated group and MI group during the 28-day observation period (data not shown). DiR-labeled HucMSC was detected in the heart, lung, liver, and spleen but absent in the kidney at 7-day postinjection, with high intensity in the lung and liver (Figure 1(b)). We also observed a small portion of HucMSC signal in the heart at 7-day post-MI (Figure 1(b)). At 28-day post-MI, the DiR signal declined in the liver and lung and was not detectable in spleen, kidney, and heart (Figure 1(b)). Interestingly, the HucMSC engraftment in the heart sequentially increased at 1-, 3-, 5-, and 7-day post-MI, preferably in the infarcted region (Figure 1(c)). We did not detect any structural change in lung, liver, spleen, and kidney in the HucMSC-treated group compared to MI and normal mice (supplementary Figure 2). More importantly, at 7- and 28-day post-MI, iv HucMSC administration markedly upregulated both LVEF and LVFS in mice subjected to cardiac infarction (Figure 1(d)). HucMSC administration reduced serum ANP and BNP levels at 7-day post-MI (Figure 1(e)). These results indicated that HucMSC could arrive at the injured heart and contributed to cardiac function recovery when administered intravenously postinfarction.

### 3.2. HucMSC Treatment Prevented Adverse Cardiac Remodeling after MI

**Figure 2:**
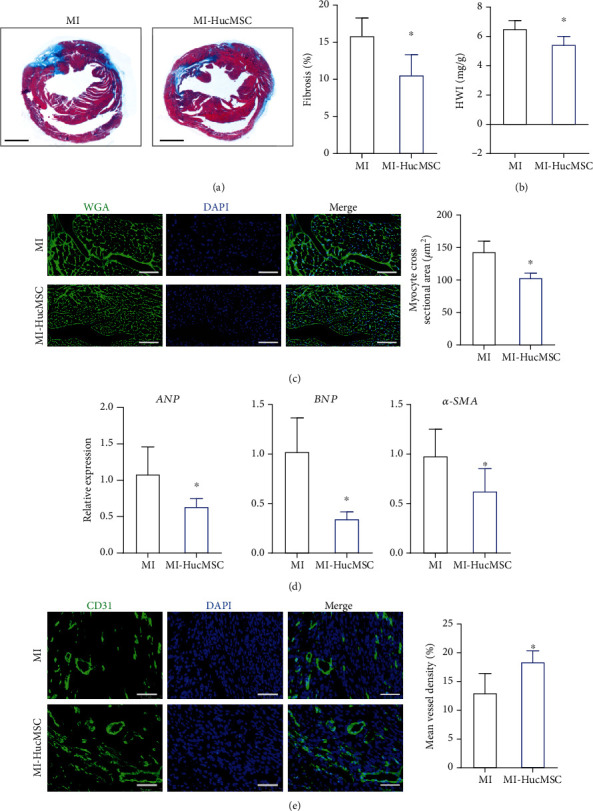
Iv-administered HucMSC prevented adverse cardiac remodeling after MI. (a) Representative images of Masson's trichrome staining and quantitative analysis of fibrosis percentage at 28 days after MI and HucMSC injection. Scale bar = 1000 *μ*m. (b) Heart weight (milligrams) to body weight (grams) ratio (HWI) at 28-day post-MI. (c) Representative images of WGA staining and quantitative analysis of cardiomyocyte CSA. Scale bar = 75 *μ*m. (d) qRT-PCR measurement of *ANP*, *BNP*, and *α-SMA* at 28-day post-MI. (e) Representative images and quantitative analysis of CD31 staining at 28-day post-MI. Scale bar = 75 *μ*m. WGA: wheat germ agglutinin; CSA: cross-sectional area. *n* = 5 ~ 6 mice for each group. ^∗^*p* < 0.05 vs. MI group.

Adverse cardiac remodeling post-MI eventually led to heart failure. Then, we evaluated whether HucMSC treatment can prevent this process in murine MI models. On day 28 post-MI, Masson's trichrome staining showed HucMSC injection reduced cardiac fibrosis compared with the control hearts (Figure 2(a)). HWI was lower in the HucMSC-treated group than in the MI group (Figure 2(b)). Moreover, cardiomyocyte cross-sectional area (CSA) was measured based on WGA staining, which revealed a reduced CSA in HucMSC-treated mice compared to MI hearts (Figure 2(c)). In addition, the mRNA levels of *ANP*, *BNP*, and *α-SMA*, which were indicative for the remodeling process, were measured by qRT-PCR. As shown in Figure 2(d), HucMSC administration significantly reduced *ANP*, *BNP*, and *α-SMA* expression compared to the MI group. In the peri-infarct area, the expression of endothelial marker CD31 was remarkably higher in the HucMSC-treated group compared to the MI control (Figure 2(e)). Collectively, these findings suggested that iv HucMSC administration prevented adverse cardiac remodeling after MI.

### 3.3. Systemic Immunomodulatory Effects of Iv-Administered HucMSC

**Figure 3:**
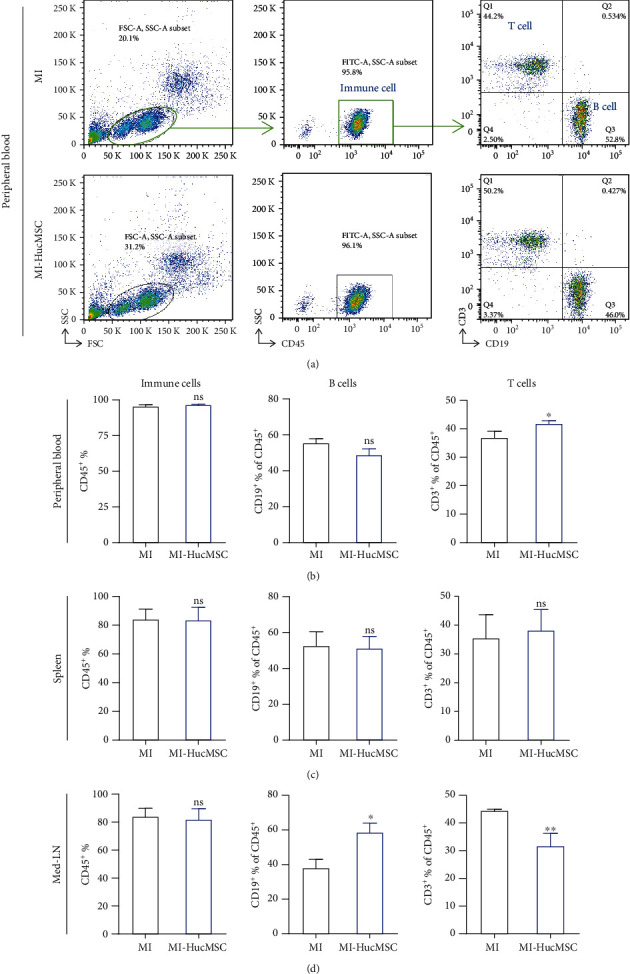
Systemic immunomodulatory effects of iv-administered HucMSC at 7-day post-MI. (a) Gating strategy and representative images of flow cytometry in the peripheral blood at 7-day post-MI. (b) The ratio of immune cells (CD45^+^%), B cells (CD19^+^% of CD45^+^), and T cells (CD3^+^% of CD45^+^) in the peripheral blood was analyzed by flow cytometry at 7-day post-MI. c) The ratio of immune cells (CD45^+^%), B cells (CD19^+^% of CD45^+^), and T cells (CD3^+^% of CD45^+^) in spleen was analyzed by flow cytometry at 7-day post-MI. (d) The ratio of immune cells (CD45^+^%), B cells (CD19^+^% of CD45^+^), and T cells (CD3^+^% of CD45^+^) in med-LNs was analyzed by flow cytometry at 7-day post-MI. *n* = 3 ~ 4 mice for each group. ns, non-significant vs. MI group; ^∗^*p* < 0.05 vs. MI group; ^∗∗^*p* < 0.01 vs. MI group.

Previous in vivo and in vitro studies proved that MSC can exert its roles through immune regulation [27, 28]. As HucMSC was administered intravenously, we first sought to determine whether HucMSC affected the inflammatory milieu in the peripheral circulatory system at 7-day post-MI. Flow cytometric analysis revealed that HucMSC injection did not alter the ratio of immune cells (CD45^+^%) or B cells (CD19^+^% of CD45^+^) but significantly increased the proportion of T cells (CD3^+^% of CD45^+^) at 7-day post-MI (Figures 3(a) and 3(b)). In spleen, there was no significant difference in the percentage of immune cells (CD45^+^%), B cells (CD19^+^% of CD45^+^), or T cells (CD3^+^% of CD45^+^) between the HucMSC and MI groups at 7-day post-MI (Figure 3(c), supplementary Figure 3A). In the med-LNs (heart draining lymph nodes), we detected a significant increase in B cell proportion (CD19^+^% of CD45^+^) and decrease in T cell proportion (CD3^+^% of CD45^+^) in the HucMSC group compared to the MI group at 7-day post-MI (Figure 3(d), supplementary Figure 3B). We further measured the protein levels of inflammatory cytokines in serum, including IFN-*γ*, IL-12p70, IL-13, IL1-*β*, IL-2, IL-4, IL-5, IL-6, TNF-*α*, GM-CSF, IL-10, IL-17A, and IL-18, at 7-day post-MI by protein array. However, most of the them, including IFN-*γ*, IL-12p70, IL-13, IL1-*β*, IL-4, GM-CSF, IL-10, IL-17A, and IL-18, were below the lower limiting level of the array in both MI and MI-HucMSC groups (data not shown). There was no significant difference in the serum expression levels of IL2, IL5, IL6, and TNF-*α* between the MI and MI-HucMSC groups (supplementary Figure 4).

### 3.4. Cardiac Immunomodulatory Effects of Iv-Administered HucMSC

**Figure 4:**
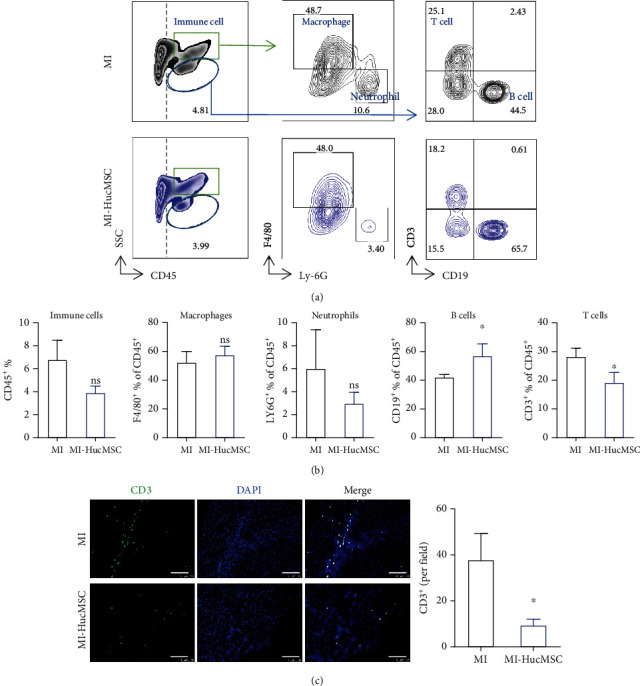
Cardiac immunomodulatory effects of iv-administered HucMSC at 7-day post-MI. (a) Gating strategy and representative images of flow cytometry in the heart at 7-day post-MI. (b) Flow cytometry analysis of immune cells (CD45^+^%), macrophages (F4/80^+^% of CD45^+^), neutrophils (Ly-6G^+^% of CD45^+^), T cells (CD3^+^% of CD45^+^), and B cells (CD19^+^% of CD45^+^) in the heart. (c) Representative images and quantitative analysis of CD3^+^ T cells in the peri-infarct area. Scale bar = 100 *μ*m. *n* = 3 ~ 4 mice for each group. ns, non-significant vs. MI group; ^∗^*p* < 0.05 vs. MI group.

Upon ischemia, the local cardiac tissue undergoes a dramatic alteration of microenvironment mainly including the changes of immune cells phenotype and function [29]. Since we monitored a small proportion of HucMSC engrafted into the injured heart (Figures 1(b) and 1(c)), we next sought to evaluate whether HucMSC could affect the local inflammatory status in the heart. At 7-day post-MI, single-cell suspensions from heart tissues were obtained, and flow cytometry results showed a downward trend in immune cells percentage (CD45^+^%) in HucMSC-treated group compared to the MI group though not statistically significant (Figures 4(a) and 4(b)). The large CD45^+^ immune cells were further gated to analyze the ratio of macrophages (F4/80^+^% of CD45^+^) and neutrophils (Ly-6G^+^% of CD45^+^) (Figure 4(a)). However, the percentage of these two cell types did not differ in the HucMSC-treated group and MI group (Figures 4(a) and 4(b)). The small CD45^+^ immune cells were gated to assess the changes of B cells (CD19^+^% of CD45^+^) and T cells (CD3^+^% of CD45^+^) (Figure 4(a)). HucMSC treatment significantly increased the proportion of B cells and decreased the proportion of T cells at 7-day post-MI (Figures 4(a) and 4(b)). Further immunostaining results confirmed the decreased CD3^+^ T cells in the HucMSC-treated hearts compared to MI hearts (Figure 4(c)).

### 3.5. Long-Term Immunomodulatory Effects of Iv-Administered HucMSC In Vivo

**Figure 5:**
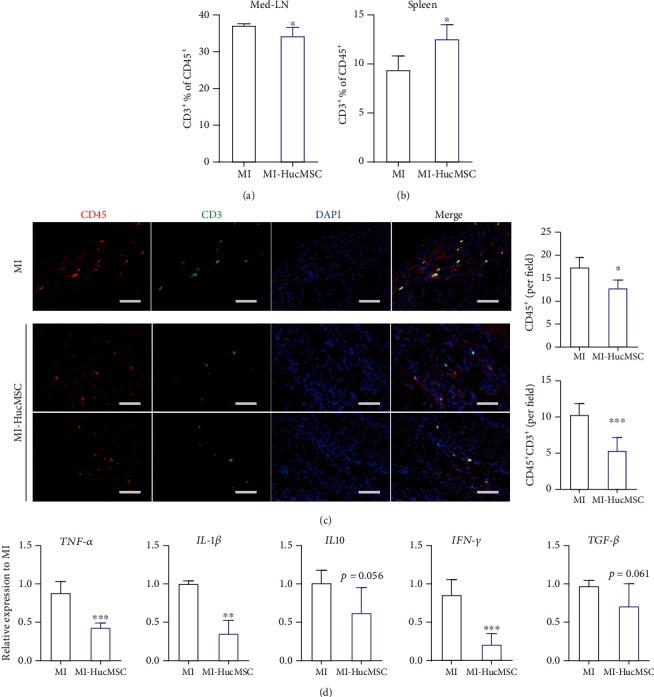
Long-term immunomodulatory effects of iv-administered HucMSC in vivo. (a) Flow cytometry analysis of T cells (CD3^+^% of CD45^+^) in the med-LNs. *n* = 3 ~ 4 mice for each group. (b) Flow cytometry analysis of T cells (CD3^+^% of CD45^+^) in the spleen. *n* = 3 ~ 4 mice for each group. (c) Representative images and quantitative analysis of CD45^+^ immune cells and CD45^+^CD3^+^ T cells in the peri-infarct area at 21-day post-MI. *n* = 5 ~ 6 mice for each group. Scale bar = 100 *μ*m. (d) qRT-PCR measurement of inflammatory cytokines in the infarcted heart. *n* = 5 ~ 6 mice for each group. ^∗^*p* < 0.05 vs. MI group; ^∗∗^p < 0.01 vs. MI group; ^∗∗∗^*p* < 0.001 vs. MI group.

To better understand the long-term immune modulatory effects of HucMSC, we measured the immune status at 21-day post-MI. HucMSC administration significantly decreased the proportion of T cells (CD3^+^% of CD45^+^) in the med-LN (Figure 5(a)), while increasing the proportion of T cells (CD3^+^% of CD45^+^) in the spleen (Figure 5(b)). In the injured heart, iv-administered HucMSC inhibited CD45^+^ immune cell infiltration compared to the MI group (Figure 5(c)). More importantly, the CD45^+^CD3^+^ T cells significantly decreased in the HucMSC-treated group compared to the MI group (Figure 5(c)). In addition, HucMSC treatment remarkably decreased the inflammatory cytokine expression, including *TNF-α*, *IL1-β*, and *IFN-γ*, in the infarcted myocardium (Figure 5(d)). Collectively, these data suggested iv-administered HucMSC after MI significantly decreased T cell infiltration and attenuated the chronic inflammatory response in the heart.

## 4. Discussion

**Figure 6:**
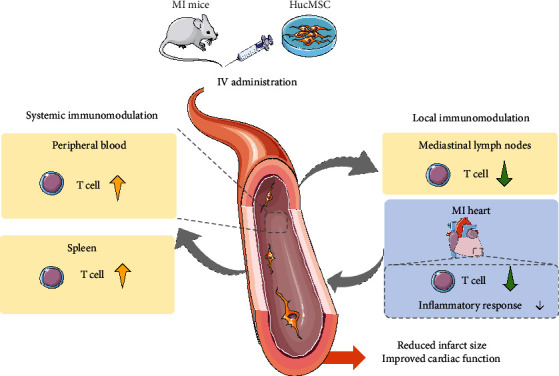
Schematic diagram. Iv-administered HucMSC attenuated cardiac function deterioration after infarction via systemic and local immune modulatory effects.

This study evaluates the efficacy of iv-administered HucMSC in treating MI from the perspective of systemic and local immune modulation and has several major findings: First, iv-administered HucMSC after MI distributed to multiple organs, including the heart, and the engrafted HucMSC in the heart persisted at least 7 days. Second, though only a small proportion of HucMSC was homed to the heart, these cells presented remarkable cardiac protective effects, as evidenced by improved LVEF and LVFS, attenuated adverse LV remodeling and increased angiogenesis after MI. Third, HucMSC-administered post-MI produced systemic immunomodulation effects in the circulating peripheral blood, med-LNs, spleen, and the infarcted heart. More importantly, iv-administered HucMSC reduced T cell infiltration and inflammatory response in the infarcted heart (Figure 6).

MSC, a heterogeneous stem cell population featured by feasible accessibility, extensive proliferation ability, multiple differentiation potential, low immunogenicity, and immunomodulation functions, is widely applied as therapeutic interventions in degenerative and/or inflammatory diseases. The therapeutic properties of MSC can be varied depending on multiple parameters, including tissue origin [30, 31]. The umbilical cord is a readily accessible source of MSC, and the HucMSC exhibits superior primitive, proliferative, and immunosuppressive capacities compared to their counterparts derived from adult sources [9, 32, 33]. In addition, emerging data suggested HucMSC was less immunogenic than BM-MSC, making them a promising alternative for allogeneic cell therapy [9]. In the present study, we evaluated the efficacy of HucMSC on cardiac protection using a murine MI model.

The action mode of MSC-mediated effects is initially believed to be via their local engraftment in the injured sites. Therefore, in most preclinical and clinical settings, intramyocardial or catheter-based delivery strategies were applied to treat cardiovascular diseases. However, these methods are invasive, and considering that LV remodeling is progressive, repeated injections might be necessary for sustained therapeutic effects. In this context, iv delivery has advantages over other invasive routes. In addition, as the excessive and prolonged inflammation gradually aggravates cardiac function after MI, iv-administered MSC could deliver systemic and local immune modulation effects, preventing cardiac functional decline after MI. Firstly, we evaluated the safety of iv administration of HucMSC in murine MI models. Our results showed that iv administration of HucMSC (30 × 10^4^ per mouse) did not significantly affect the survival during the 28-day post-MI observation period (data not shown). Furthermore, there were no distinct structural alterations in the main organs where most of the HucMSC retained, suggesting iv-administered HucMSC was potentially safe as a therapeutic approach to treat MI.

Previous studies indicated iv-administered BM-MSC was mostly trapped in the lungs where they encountered sequestration, with a small proportion of cells accumulated in the heart [2, 34]. Our data was partially in line with the previous findings. We showed iv-administered HucMSC was predominately concentrated in the lung, spleen, and liver, with a small proportion of cells engrafted in the heart (Figure 1(b)). We detected the fluorescent intensity of DiR-labeled HucMSC sequentially increased at 1-, 3-, 5-, 7-day post-MI, preferably in the infarcted region, indicating HucMSC responded dynamically to the signal released from the infarcted heart (Figure 1(c)). The biodistribution of iv-administrated HucMSC after infarction indicated that these cells might exert effects systemically and locally.

Previously, Luger et al. showed iv-administered human BM-MSC, in a dose of 100 × 10^4^ per mouse, prevented the adverse LV remodeling and functional decline in a mouse model of ischemia and reperfusion [2]. In the present study, iv administration of HucMSC, with a dose of 30 × 10^4^ per mouse, was effective in alleviating adverse LV remodeling, as well as improving cardiac functional performance and angiogenesis after MI. A possible reason for the discrepancies between our data and Luger et al. could be the different sources of MSC. It is reported iv-administered HucMSC persisted longer than BM-MSC, possibly due to their surface proteins, allowing some of the cells to escape the lungs [35]. Therefore a relatively lower dosage of HucMSC administered after infarction attained a beneficial therapeutic value.

Previous studies revealed the immune modulatory effects of MSC and their derivatives in treating wound healing [36], autoimmune encephalomyelitis [37], and COVID-19 pneumonia [38] after iv administration. We recently demonstrated that a cardiac injury signal could trigger systemic inflammation through the body [39]. Moreover, ischemic injury mobilizes a diverse repertoire of innate and adaptive immune cells [40]. In the current study, we determined HucMSC administration via the iv route exerted a systemic immune modulation action, in which the CD3^+^ T cell ratio in the peripheral blood increased in HucMSC-treated mice at 7-day post-MI (Figures 3(a) and 3(b)). Interestingly, CD3^+^ T cell proportion significantly decreased in the med-LNs as well as in the infarcted heart (Figures 3(d) and 4), suggesting HucMSC-mediated interchange of CD3^+^ T cells in the injured heart, its draining med-LNs, and the periphery. Notably, the inhibition of iv-administered HucMSC on cardiac T cell infiltration was sustained to 21-day post-MI (Figure 5(c)), with the same trend observed in the adjacent med-LN (Figure 5(a)). After infarction, T lymphocytes become activated and proliferate in the med-LNs and then infiltrate the myocardium [41]. Prolonged T cell activation in the diseased heart contributes to the progression of pathological LV remodeling [42]. Adoptive transfer of cardiac CD3^+^ T cells from donor mice with heart failure induced LV dysfunction, fibrosis, and hypertrophy in naïve recipient mice [42]. Our results suggested iv HucMSC administration might serve as an alternative to suppress sustained T cell response in the infarcted heart. In addition, HucMSC suppressed inflammatory cytokine expression in the infarcted heart (Figure 5(d)). Our results indicated that iv-administrated HucMSC may contribute to cardiac function recovery partly through regulation of the local immune responses.

The spleen exerts an obligatory role in the progression of cardiac remodeling and inflammation by responding to the injured signal and altering its immune cell populations accordingly [43]. In the present study, HucMSC treatment did not alter splenic CD19^+^ B cell and CD3^+^ T cell at 7-day post-MI (Figure 3(c)). However, the treatment of HucMSC increased CD3^+^ T lymphocytes in the spleen at 21-day post-MI (Figure 5(b)). Considering that HucMSC inhibited CD3^+^ T cell population in the infarcted heart and med-LNs (Figures 3(d), 4, and 5), the efficacy of iv-administered HucMSC on cardiac function may partly attribute to their control of the cardio-splenic axis post-MI.

The current study still has some limitations. First, with stem cell-based therapy, proper dosage, timing, and delivery route have historically been a complicated and vexed question. Here we showed that iv-administered HucMSC dosing 30 × 10^4^ after acute MI had considerable therapeutic values. However, whether iv-administered HucMSC has a dose-dependent effect on cardiac performance, or whether HucMSC administered at chronic phase of MI still exert cardiac protection, or whether iv route has a comparable therapeutic effect compared to intramyocardial injection—these questions need further investigations. Second, we used human umbilical cord as MSC source as HucMSC display greater proliferative and immunosuppressive capacities compared to those derived from adult tissues. However, adult MSC from different sources or different donors contribute to cell heterogeneity. Alternatively, induced pluripotent stem cell (iPSC) might serve as a robust source of MSC. Several groups published accessible method to derive MSC from iPSC (iMSC) [14, 44, 45]. More importantly, iMSC and their by-products, such as conditioned medium and extracellular vesicles, displayed high potency in immunomodulatory properties and regenerative potential [46–49]. iMSC might represent one of the future perspectives in MSC-based therapy. Comparison of HucMSC and iMSC in treating cardiovascular diseases might provide practical information for future clinical applications. Third, MSC exerted their roles via multiple mechanisms. Our present study mainly focused on the local and systemic immune regulation. Whether iv administration of HucMSC acts in the injured heart in other ways needs further exploration.

Collectively, our study suggested iv-administered HucMSC-mediated lymphocyte exchange between the peripheral blood, spleen, med-LNs, and the infarcted hearts following infarction. The HucMSC-mediated systemic and local immunomodulatory effects play a role in the MSC-induced improvement in LV function and adverse LV remodeling after MI.

## Data Availability

All data and materials are available on request.

## Additional Points

*Highlights*. (1) Intravenous administration of HucMSC engrafted in the murine heart and persisted at least 7 days after MI. (2) HucMSC treatment improved LVEF and LVFS, attenuated adverse LV remodeling, and increased angiogenesis in murine MI models. (3) HucMSC administration post-MI produced systemic immunomodulation and reduced T cell infiltration into the injured heart after MI.

## Supplementary Materials

Supplementary Materials Supplementary Figure 1: characterization of HucMSC. A) Surface marker profile of HucMSC at passage 4. B) Trilineage differentiation capacity (adipogenesis, osteogenesis, chondrogenesis) of HucMSC at passage 4. Supplementary Figure 2: representative hematoxylin-eosin staining images of main organs at 28 days after MI and HucMSC administration. *n* = 3 ~ 4 mice for each group. Scale *bar* = 200 *μ*m. Supplementary Figure 3: A) gating strategy and representative images of flow cytometry in the spleen at 7-day post-MI. B) Gating strategy and representative images of flow cytometry in the med-LN at 7-day post-MI. Supplementary Figure 4: serum levels of IL2, IL5, IL6, and TNF-*α* at 7-day post-MI were measure by protein array. *n* = 3 ~ 4 mice for each group. ns, non-significant vs. MI group.
