# Supplementary material for: Intravenously Administered Human Umbilical Cord-Derived Mesenchymal Stem Cell (HucMSC) Improves Cardiac Performance following Infarction via Immune Modulation
Source: Stem Cells Int. 2023 Mar 17;2023:6256115. doi: 10.1155/2023/6256115 (PMC10038737; doi:10.1155/2023/6256115)

**supplementary information**

**Intravenously administered** **human umbilical cord-derived mesenchymal stem cell (HucMSC) improves cardiac performance following infarction via immune modulation**

Xiaoting Liang^†1, 2^, Jing Liu^†1, 2, 3^, Mimi Li^1, 4^, Fang Lin^1, 4^, Rulin Zhuang^1, 5^, Qingshu Meng^1, 4^, Xiaoxue Ma^1, 4^, Yuanfeng Xin^1, 5^, Xin Gong^6, 7^, Zhiying He^2, 7^, Wei Han^6, 7^, Xiaohui Zhou^*1, 4^, Zhongmin Liu^*1, 4, 5, 7^

^†^Dr. Liang and Dr. Liu contributed equally to this work.

1. Research Center for Translational Medicine, Shanghai East Hospital, Tongji University School of Medicine, Shanghai 200120, P.R. China;
2. Institute for Regenerative Medicine, Shanghai East Hospital, School of Life Sciences and Technology, Tongji University, Shanghai 200120, P.R. China;
3. Department of Burn & Plastic Surgery, Beijing Children's Hospital, Capital Medical University, National Center for Children's Health, Beijing, 100045, P.R. China;
4. Shanghai Heart Failure Research Center, Shanghai East Hospital, Tongji University School of Medicine, Shanghai 200120, P.R. China;

5. Department of Cardiovascular Surgery, Shanghai East Hospital, Tongji University School of Medicine, Shanghai 200120, P.R. China;

6. Department of Heart Failure, Shanghai East Hospital, Tongji University School of Medicine, Shanghai 200120, P.R. China;

7. Shanghai Institute of Stem Cell Research and Clinical Translation, Shanghai 200120, China

* To whom correspondence should be addressed:

Xiaohui Zhou, M.D., Ph.D.

Research Center for Translational Medicine,

Shanghai East Hospital, Tongji University School of Medicine

150 Jimo Rd, Pudong, Shanghai, 200120, China

Tel:86-21-61569884

E-mail: zxh100@tongji.edu.cn

Zhongmin Liu, M.D., Ph.D.

Research Center for Translational Medicine,

Department of Cardiovascular Surgery,

Shanghai East Hospital, Tongji University School of Medicine

150 Jimo Rd, Pudong, Shanghai, 200120, China

E-mail: [liu.zhongmin@tongji.edu.cn](mailto:liu.zhongmin@tongji.edu.cn)

**Figure legend**

**supplementary Figure 1.** Characterization of HucMSC. A) Surface marker profile of HucMSC at passage 4. B) Trilineage differentiation capacity (adipogenesis, osteogenesis, chondrogenesis) of HucMSC at passage 4.

**supplementary Figure 2.** Representative hematoxylin-eosin staining images of main organs at 28 days after MI and HucMSC administration. n = 3~4 mice for each group. scale bar = 200 μm.

**supplementary Figure 3.** A) Gating strategy and representative images of flow cytometry in the spleen at 7 days post-MI. B) Gating strategy and representative images of flow cytometry in the med-LN at 7 days post-MI.

**supplementary Figure 4.** Serum levels of IL2, IL5, IL6 and TNF-α at 7 days post MI were measure by protein array. n = 3~4 mice for each group. ns, non-significant vs MI group.


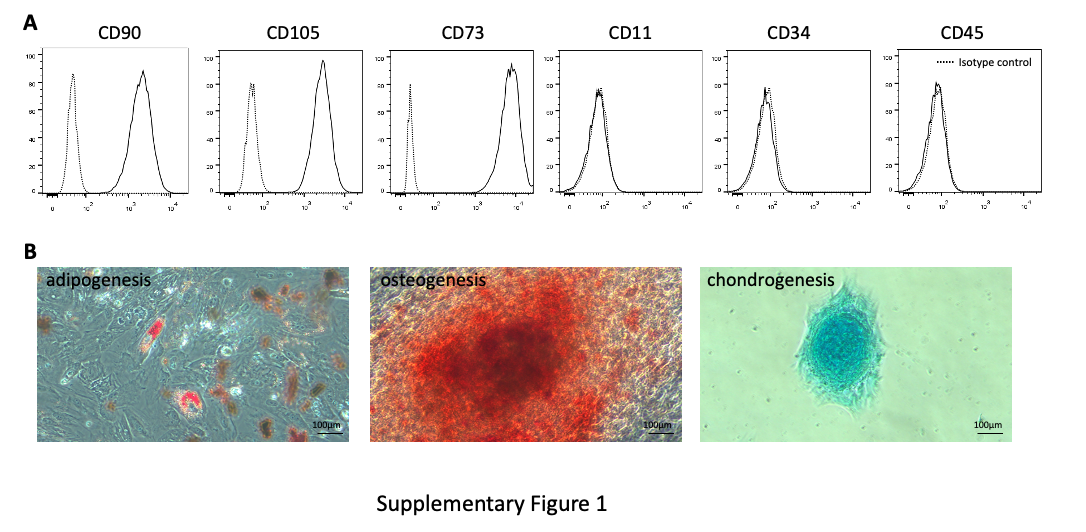


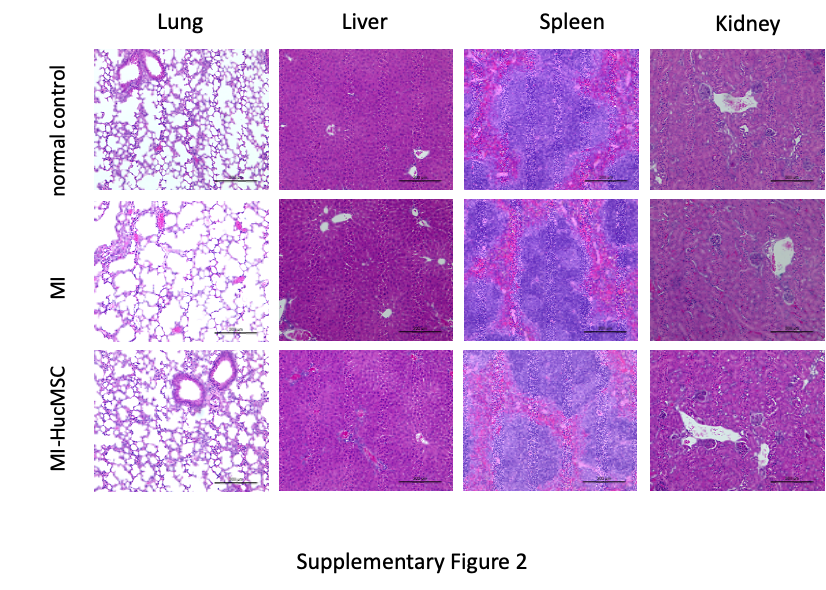


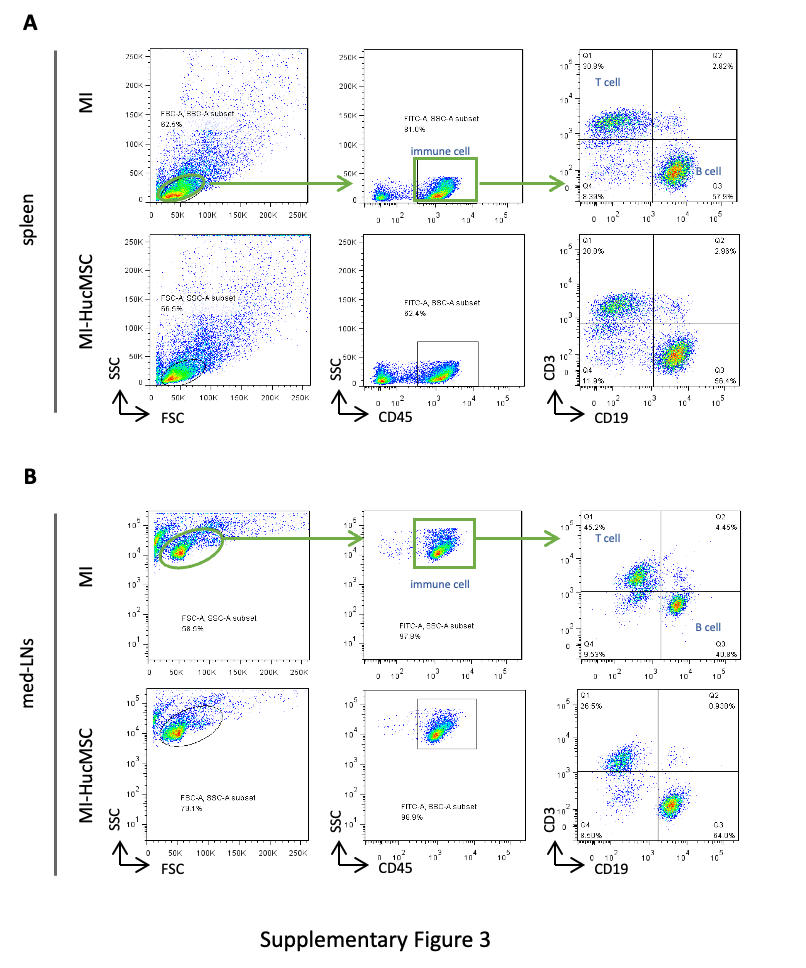


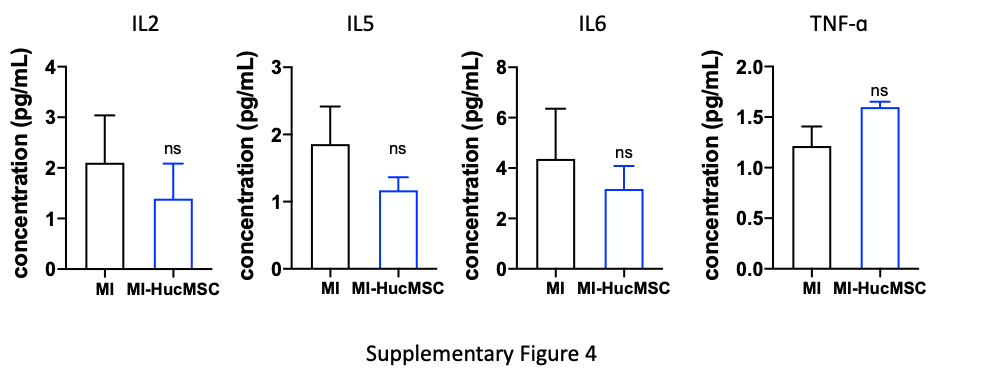

Supplement: Supplementary Materials — Supplementary Figure 1: characterization of HucMSC. A) Surface marker profile of HucMSC at passage 4. B) Trilineage differentiation capacity (adipogenesis, osteogenesis, chondrogenesis) of HucMSC at passage 4. Supplementary Figure 2: representative hematoxylin-eosin staining images of main organs at 28 days after MI and HucMSC administration. n = 3 ~ 4 mice for each group. Scale bar = 200 μm. Supplementary Figure 3: A) gating strategy and representative images of flow cytometry in the spleen at 7-day post-MI. B) Gating strategy and representative images of flow cytometry in the med-LN at 7-day post-MI. Supplementary Figure 4: serum levels of IL2, IL5, IL6, and TNF-α at 7-day post-MI were measure by protein array. n = 3 ~ 4 mice for each group. ns, non-significant vs. MI group. [file 6256115.f1.docx]
